# Usutu virus NS4A induces autophagy and is targeted by the selective autophagy receptor p62/SQSTM1 for degradation

**DOI:** 10.1186/s12985-025-02719-5

**Published:** 2025-04-17

**Authors:** Tessa Nelemans, Ali Tas, Nina L. de Beijer, George M. C. Janssen, Peter A. van Veelen, Martijn J. van Hemert, Marjolein Kikkert

**Affiliations:** 1 Molecular Virology Laboratory, Center for Infectious Diseases (LUCID), Leiden University, Leiden University Medical Center, Albinusdreef 2, Leiden, 2333 ZA The Netherlands; 2 Center for Proteomics and Metabolomics, Leiden University Medical Center, Leiden, The Netherlands

**Keywords:** Usutu virus, Orthoflavivirus, NS4A, Autophagy, p62, SQSTM1

## Abstract

Usutu virus (USUV) is an emerging orthoflavivirus, which mainly affects birds but in rare cases can cause severe neuroinvasive disease in humans. The virus relies on a multitude of host cell proteins, molecules and cellular processes for its replication, and must subvert host antiviral responses to establish a successful infection. Studying the complex network of virus-host protein interactions by proteomics approaches can therefore provide new insights in the replication cycle of USUV and its pathogenesis. We have previously shown that the USUV protein NS4A acts as an antagonist of the antiviral interferon response, and here we further map the host interaction partners of USUV NS4A using proximity labeling coupled to mass spectrometry. The resulting NS4A interactome revealed many host proteins involved in the autophagy pathway. We showed that both USUV infection and overexpression of USUV NS4A can indeed induce the autophagy pathway. However, stimulation or inhibition of the autophagy pathway in general did not affect USUV replication. Therefore, we decided to specifically analyze the role of the selective autophagy receptor sequestosome 1 (p62/SQSTM1), since we identified this protein as an important interaction partner of USUV NS4A. We found that p62 is involved in the degradation of USUV NS4A. In agreement with this, the knockdown of p62 enhanced replication of USUV in A549 cells. P62 thus plays an antiviral role during USUV infection, although this antiviral effect might also be related to its functions outside the autophagy pathway, such as modulation of the immune response. In conclusion, this study showed that USUV NS4A induces autophagy and is then targeted by p62 for degradation by the autophagic machinery, uncovering a new role of p62 in the antiviral defense against USUV.

**Supplementary Information:**

The online version contains supplementary material available at 10.1186/s12985-025-02719-5.

## Introduction

Usutu virus (USUV) is an emerging orthoflavivirus that originates from Africa, and is now endemic in several European countries and expanding further globally [1, 2]. USUV is transmitted by *Culex* mosquitos and mainly circulates between birds and mosquitos, but it can occasionally be transmitted by infected mosquitos to other vertebrate hosts including humans. USUV can cause high mortality in certain bird species (like blackbirds), but can also cause disease in humans (with underlying conditions). USUV has the potential to be neuroinvasive similar to West Nile virus (WNV), and human cases of neurological disease caused by USUV have been reported [1, 3].

The USUV genome consists of a positive-sense single-stranded RNA genome of around 11 kb which is translated into one large polyprotein. This polyprotein is cleaved by host and viral proteases into 3 structural (capsid, membrane, envelope) and 7 nonstructural (NS) proteins (NS1, NS2A, NS2B, NS3, NS4A, NS4B, NS5) [3, 4]. The structural proteins are involved in virus binding, fusion, and entry during infection and biogenesis and maturation of virions at the final stages of the replication cycle. The nonstructural proteins are essential for replication of the viral genome, but are also involved in assembly and are important for modulation of the host immune response [4]. Because of the limited number of proteins encoded by orthoflaviviruses, the virus needs to rely on many proteins, metabolites, organelles, and processes of the host cell to successfully replicate. At the same time viruses have to counteract the antiviral immune response in order to create their window-of-opportunity. This means that the virus interacts with many host proteins to ‘remodel’ the cell in order to support virus replication [5]. Proteomic approaches are a powerful way to obtain insight into virus-host protein-protein interactions [6]. These hypothesis-free proteome-wide approaches have resulted in interactome data sets for several orthoflaviviruses including Dengue virus (DENV), Zika virus (ZIKV) and WNV [7–10]. However, no interactome data is available yet for USUV, and only a limited number of studies identified specific interactions between USUV proteins and individual host factors [11, 12].

**Fig. 1:**
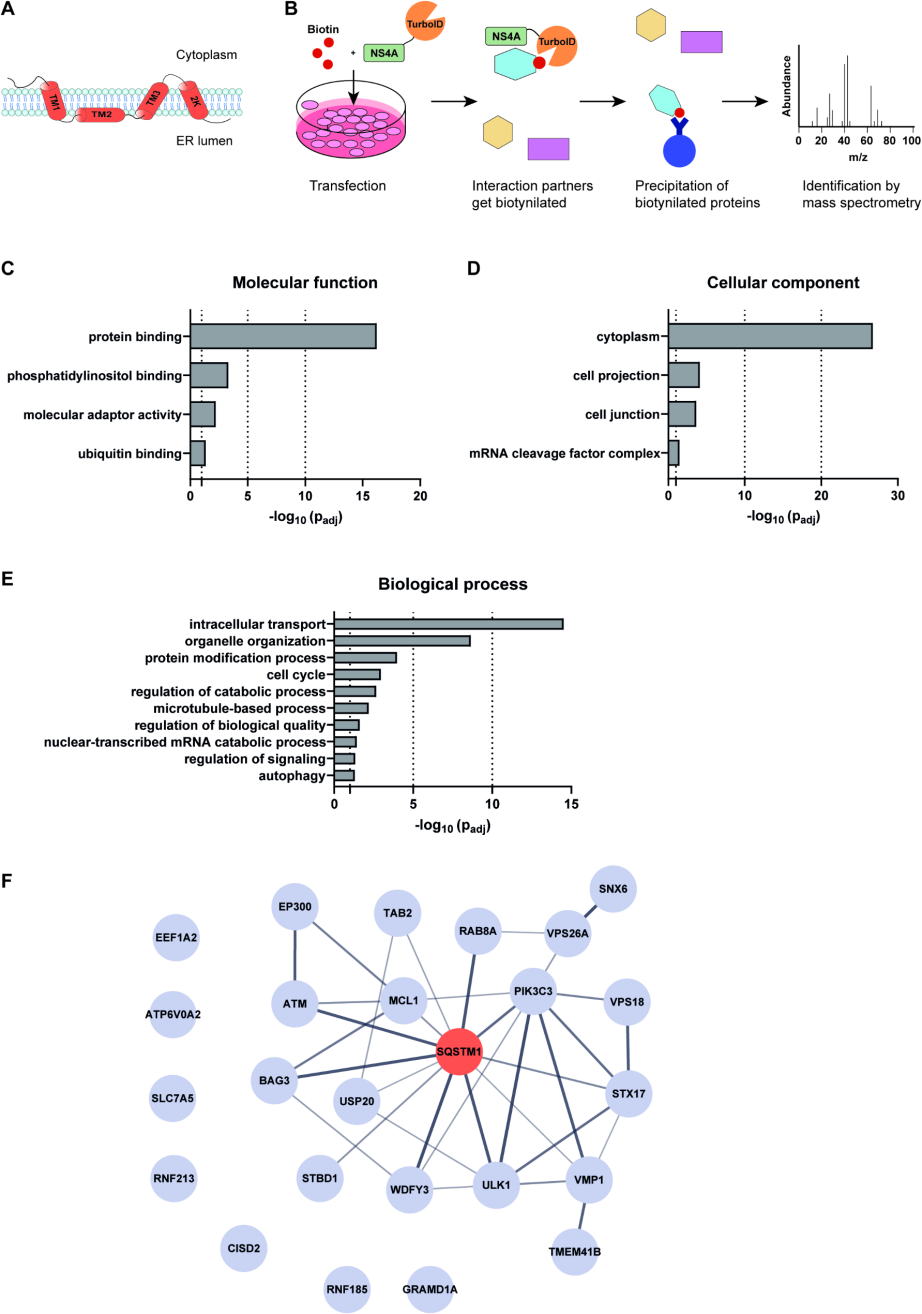
The USUV NS4A interactome is enriched for autophagy related proteins. **A** Schematic representation of USUV NS4A structure. **B** Schematic overview of the workflow of the proximity labeling assay. **C-E** Gene ontology analysis of the USUV NS4A interactome to identify statistically enriched molecular functions (**C**), cellular components (**D**), and biological processes (**E**). **F** STRING network analysis of potential interaction partners of USUV NS4A as identified in the proximity screen involved in autophagy. Line thickness indicates the degree of confidence prediction of the interaction (ranging from 0.4 to 1). The interaction of SQSTM1/p62 (orange) with USUV NS4A was further validated in this study

In this study we set out to map the potential host interaction partners of USUV NS4A, a transmembrane protein (Fig. 1A) that mainly localizes to the ER. The function of USUV NS4A remains poorly characterized, but NS4A proteins of other orthoflaviviruses have been shown to be involved in the rearrangement of membranes, the unfolded protein response, autophagy, and immune evasion [13]. We recently showed that USUV NS4A can functions as an antagonist of the interferon (IFN) response [12] and we now further characterized the functions of USUV NS4A by identifying the USUV NS4A interactome. We used proximity labeling coupled to mass spectrometry to identify interaction partners of NS4A. This dataset revealed that the interacting proteins were enriched (among others) for the autophagy pathway.

Autophagy is a process of protein and organelle degradation important for maintaining cellular homeostasis, which can exert both proviral and antiviral effects [14]. During macroautophagy cytoplasmic components get encapsulated in double-membrane vesicles: the autophagosomes. During the elongation and maturation of the autophagosomes, microtubule-associated protein 1 light chain 3 (LC3) is cleaved into LC3-I and then conjugated to phosphatidylethanolamine (PE) resulting in LC3-II, which is incorporated in autophagosome membranes [15]. The mature autophagosome then fuses with lysosomes and the content gets degraded and recycled [16, 17]. Some orthoflaviviruses such as DENV and ZIKV can induce autophagy and it is thought that this enhances their replication [18]. On the other hand, other orthoflaviviruses do not induce autophagy, or are even restricted by activation of the autophagy pathway [18]. We show here that USUV NS4A can induce autophagy, but found that the induction of autophagy does not influence USUV replication per se. We then focused in more detail on the role of one of the identified interaction partners from NS4A: sequestosome 1 (p62/SQSTM1), a selective autophagy cargo receptor [19]. In this study, we show that p62 is involved in the autophagic degradation of USUV NS4A and plays a role in the antiviral defense against USUV.

## Materials and methods

### Cell lines

A549, BHK-21J [20], Vero CCL-81, Huh7 (Japanese Collection of Research Bioresources), H1299/ACE2 [21] and 293T (ATCC CRL-3216) cells were all grown at 37 °C in a 5% CO_2_ incubator. A549 and Vero CCL-81 cells were maintained in Dulbecco’s modified Eagle’s medium (DMEM, Gibco) supplemented with 8% fetal calf serum (FCS, Capricorn Scientific), and 50 units/mL of penicillin/streptomycin (p/s, Sigma-Aldrich). H1299/ACE2 cells were maintained in DMEM containing 10% FCS and p/s that was also supplemented with 1200 µg/mL G418 every third passage. Huh7 cells were maintained in DMEM supplemented with 8% FCS, 2 mM L-glutamine (Sigma), MEM non-essential amino acids (Gibco), and 50 units/mL of p/s. 293T cells were grown in DMEM supplemented with 10% FCS, 2 mM L-glutamine, and 50 units/mL of p/s. BHK-21 J cells were cultured in Glasgow’s MEM (GMEM, Gibco) supplemented with 8% FCS, 10% tryptose phosphate broth (Gibco), 10 mM HEPES (Lonza), and 50 units/mL of p/s.

Mouse embryonic fibroblasts (MEFs) were cultured in DMEM supplemented with 10% FCS, and 50 units/mL p/s at 37 °C in a 5% CO_2_ incubator. ATG3−/− MEFs and the control wildtype cells were kindly provided by Prof. Masaaki Komatsu (Tokyo Metropolitan Institute Medical Science) [22]. ATG5−/− MEFs and the control wildtype cells were provided by Prof. Noboru Mizushima (University of Tokyo) [23]. ATG13−/− MEFs and the wild type control cells were provided by Prof. Xiaodong Wang (Beijing National Institute of Biological Sciences) [24]. MEFs reconstituted with ATG3 and ATG5 genes were provided by Dr. Vera Kemp and Prof. Rob Hoeben (LUMC) [25]. MEFs reconstituted with the Atg13 gene were provided by Dr. Fulvio Reggiori (UMCG) [26].

### Plasmids

The sequence encoding the USUV nonstructural protein 4A was amplified by PCR using a full-length cDNA clone (based on USUV Netherlands 2016 strain, GenBank: MH891847) as template. The PCR product was cloned into the mammalian expression vectors 2HA-TurboID-N1 or 2HA-TurboID-C2 [27, 28] by standard restriction enzyme based methods. The expression constructs of the USUV nonstructural proteins have been described previously [12]. pEGFP-LC3B was kindly provided by Karla Kirkegaard [29]. The p62-HA plasmid [30] was obtained from Addgene (#28027) and it was modified by replacing the HA tag with a C-terminal FLAG tag. The p62-ΔLIR plasmid was generated by amplifying specific regions of the full-length p62 construct using PCR, and assembly of the resulting two PCR fragments into the vector using the NEBuilder^®^ HiFi DNA Assembly Cloning Kit. All primers used for construction of plasmids are listed in table S1.

### Viruses, plaque assay and compounds

The USUV isolate used in this study was the Netherlands 2016 strain (lineage Africa 3, GenBank: MH891847.1) [31]. Virus stocks were grown in Vero CCL-81 cells until passage 4 and titrated in BHK-21 J cells by plaque assay.

Plaque assays were performed by making 10-fold dilutions of virus samples in DMEM with 2% FCS. The virus dilutions (0,25 mL) were then used to infect BHK-21 J cells in 12-wells clusters and the clusters were incubated at 37 °C on a shaker. After 1 h, the inoculum was replaced with overlay medium (DMEM with 1.2% Avicel (FMC BioPolymer), 1% p/s, 2% FCS, and 50 mM HEPES). After 4 days, cells were fixed with 3.7% formaldehyde in PBS and plaques were visualized by crystal violet staining.

For infection experiments, USUV dilutions were made in DMEM with 2% FCS and p/s. Cells were infected for 1 h at 37 °C at the indicated MOIs. After removal of the inoculum, cells were washed three times with PBS and they were incubated with medium (DMEM containing 3% FCS, 2 mM L-glutamine, 20 mM HEPES, 0.075% Sodium Bicarbonate (Lonza), and 50 units/mL of p/s) at 37 °C until the moment of harvesting. Supernatants for infectious virus titer determination and/or cell lysates for RNA or protein analysis were collected. Virus titers were determined by plaque assay as described above. To study the effect of the autophagy pathway on virus replication, cells were treated with rapamycin (1 µM), wortmannin (1 µM) or bafilomycin A1 (100 nM) after the 1-hour virus adsorption period. Rapamycin, wortmannin, and bafilomycin A1 were purchased from MedChemExpress.

### Proximity-based biotinylation assay

293T cells (in 6-cm dishes) were transfected using PEI 25KT with the indicated NS4A constructs or an empty vector as control. At 24 h post transfection (hpt) cells were treated with biotin (Sigma) at a final concentration of 50µM for 1 h. Next, the cells were washed twice with PBS and harvested in lysis buffer (0.5% NP-40, 50 mM Tris-HCl pH 8.0, 150 mM NaCl, 5 mM MgCl, supplemented with cOmplete protease inhibitors (Roche)). Protein lysates were incubated for 20 min at 4 °C while rotating, and then cleared by centrifugation (20,000 x g for 15 min at 4 °C). The cleared supernatant was used for immunoprecipitation with Pierce High Capacity NeutrAvidin Agarose (Thermo Scientific). In short, pre-washed NeutrAvidin beads were added to the lysates and incubated overnight at 4 °C with gentle agitation. Next, the beads were washed four times with lysis buffer containing 1% SDS. The proteins were eluted from the beads using 2x LDS (Thermo Scientific) containing DTT.

### Mass spectrometry

Gel slices were first washed 3x with water, and subsequently subjected to reduction with 10 mM dithiothreitol, alkylation with 50 mM of iodoacetamide, and in-gel trypsin digestion using a Proteineer DP digestion robot (Bruker). After addition of trypsin (at 12.5 ng/ul) and swelling of the bands, the bands were transferred to Eppendorf vials and the bands were covered in 25 mM NH_4_HCO_3_ pH 8.3. Tryptic digestion took place overnight at 37 °C and the peptides were extracted from the gel slices with 50/50/0.1 v/v/v water/acetonitril/formic acid. Finally peptides were lyophilized.

Peptides were dissolved in 0.1% formic acid and subsequently analyzed by on-line C18 nanoHPLC MS/MS with a system consisting of an Ultimate3000 nano gradient HPLC system (Thermo, Bremen, Germany), and an Exploris480 mass spectrometer (Thermo). Samples were injected onto a cartridge precolumn (300 μm × 5 mm, C18 PepMap, 5 μm, 100 A), and eluted via a homemade analytical nano-HPLC column (30 cm × 75 μm; Reprosil-Pur C18-AQ 1.9 μm, 120 A (Dr. Maisch, Ammerbuch, Germany)). The gradient was run from 2 to 40% solvent B (20/80/0.1 water/acetonitrile/formic acid (FA) v/v/v) in 120 min. The nano-HPLC column was drawn to a tip of ∼10 μm and acted as the electrospray needle of the MS source. The mass spectrometer was operated in data-dependent MS/MS mode for a cycle time of 3 s, with a HCD collision energy at 30% and recording of the MS2 spectrum in the orbitrap, with a quadrupole isolation width of 1.2 Da. In the master scan (MS1) the resolution was 120,000, the scan range 400–1500, at standard AGC target @maximum fill time of 50 ms. A lock mass correction on the background ion m/z = 445.12 was used. Precursors were dynamically excluded after *n* = 1 with an exclusion duration of 10 s, and with a precursor range of 10 ppm. Charge states 2–5 were included. For MS2 the first mass was set to 110 Da, and the MS2 scan resolution was 30,000 at an AGC target of “standard” with a maximum fill time set t0 60 ms.

In a post-analysis process, raw data were first converted to peak lists using Proteome Discoverer version 2.2 (Thermo Electron), and submitted to the Uniprot database (Homo sapiens, 20596 entries), using Mascot v. 2.2.07 (www.matrixscience.com) for protein identification. Mascot searches were with 10 ppm and 0.02 Da deviation for precursor and fragment mass, respectively, and trypsin as enzyme. Up to two missed cleavages were allowed. Methionine oxidation and acetyl on protein N-terminus were set as a variable modification; carbamidomethyl on Cys, TMTpro on N-terminus and Lys were set as a fixed modification. Protein FDR was set to 1%. Normalization was on total peptide amount.

### Network analysis

We selected 391 highly enriched proteins for further analysis. We included proteins that had a significantly enriched abundance ratio (adjusted p-value < 0.01) in both the NS4A samples. P-values of the abundance ratios were computed using the background-based hypothesis test in Proteome Discoverer. Gene ontology enrichment analysis of the list of 391 enriched proteins was done using g: Profiler (version e111_eg58_p18_f463989d). Plotted were the driver GO terms from the ‘Molecular function’, ‘Biological processes’, and ‘Cellular components’ categories. Protein-protein interactions were visualized using the stringApp in Cytoscape (version 3.9.1).

### Western blotting

For western blot analysis cells were lysed in RIPA buffer (1% IGEPAL, 0.1% SDS, 0.5% deoxycholate, 150mM NaCl, 20mM Tris-HCl pH 7.5) supplemented with cOmplete protease inhibitors (Roche). Lysates were cleared by centrifugation (15,000 x g for 15 min at 4 °C) and protein concentrations were measured by Pierce BCA Protein Assay Kit (Thermo Scientific). Equal amounts of protein were separated by sodium dodecyl sulfate-polyacrylamide gel electrophoresis (SDS-PAGE) and then transferred onto a PVDF membrane (Merck Millipore). The membranes were blocked in 5% dried milk powder in PBS with 0.05% Tween-20 (PBST) for at least 1 h. The blots were then incubated with the primary antibodies in PBST + 5% BSA overnight at 4 °C. The following primary antibodies were used: mouse anti-HA (clone HA.C5, Abcam), mouse anti-V5 (clone 2F11F7, Thermo Fisher/Invitrogen), rabbit anti-FLAG (clone D6W5B, Cell Signaling Technology), mouse anti-LC3B (clone 8E10, MBL Life Science), rabbit anti-p62/SQSTM1 (polyclonal, MBL Life Science), rabbit anti-ATG5 (EPR1755(2), Abcam), rabbit anti-ATG13 (SAB4200100, Sigma-Aldrich), mouse anti-vinculin (clone 2B5A7, Proteintech), and rabbit anti-GAPDH (clone 14C10, Cell Signaling Technology). Lastly, the membranes were incubated with a horseradish peroxidase-conjugated secondary antibody for 1 h. The blots were visualized with Clarity Western ECL Substrate (Bio-rad). Quantification of the band intensities was done using NineAlliance software (v18.12, Uvitec).

### Immunofluorescence assay

H1299 or Huh7 cells were grown on glass coverslips. H1299 cells were transfected with plasmids encoding the individual USUV nonstructural proteins and LC3-GFP using PEI 25KT. Huh7 cells were either infected as described above or treated with rapamycin (1 µM), a combination of rapamycin and wortmannin (1 µM), or bafilomycin A1 (100 nM). At 24 h after transfection, treatment, or infection, the cells were fixed in 3% paraformaldehyde diluted in PBS. Staining was performed as described previously [12]. In brief, cells were permeabilized with PBS containing 0.1% Triton and then incubated with anti-LC3 (polyclonal, MBL Life Science), anti-pan-flavi-Env antiserum (4G2, Novus Biologicals), anti-HA (clone HA.C5, antibodies.com), or anti-V5 (clone V5.E10, antibodies.com) diluted in PBS with 5% FCS. The coverslips were washed and incubated in the dark with donkey anti-rabbit IgG conjugated to Cy3 (Jackson ImmunoResearch Laboratories) or goat anti-mouse IgG conjugated to Alexa 488 (Thermo Fisher). Nuclei were visualized with Hoechst 33,258 (Thermo Fisher). Images were taken using a Zeiss Axioskop 2 fluorescence microscope and Axiovision software (v4.7).

### Immunoprecipitation assay

293T cells (in 10-cm dishes) were transfected using PEI 25KT with pCAGGS-USUV-NS4A and p62-FLAG. At 24 hpt cells were washed once with PBS and incubated for 15 min at 4 °C in IP lysis buffer (20 mM Tris-HCl pH 7.4, 135 mM NaCl, 1% Triton X-100, 10% glycerol supplemented with cOmplete protease inhibitors (Roche)). Lysates were cleared by centrifugation (10,000 x g for 10 min at 4 °C) and then incubated with Pierce™ DYKDDDDK Magnetic Agarose (Thermo Scientific) overnight at 4 °C with gentle agitation. Using a magnetic rack, the beads were washed three times with wash buffer (0.3 M NaCL, 0.1% Triton X-100 in PBS), and once with water. The beads were boiled in 2x Laemmli sample buffer to elute the proteins. Proteins were separated by SDS-PAGE and analyzed by western blot as described above, except the detection was performed using a three-step protocol. In short, after a 1-hour incubation with a biotin-conjugated secondary antibody, the membranes were incubated with a Cy3-conjugated mouse-anti-biotin antibody (Jackson Laboratories) for 1 h in the dark before a fluorescent image was captured on the Uvitec system.

### SiRNA transfection

A549, Huh7, or 293T cells (in 24-wells or 12-wells plates) were transfected using Dharmafect 1 (Horizon) with siRNA targeting p62 (human SQSTM1 siGENOME SMARTpool, Horizon Discovery) at a final concentration of 10 nM, following the manufacturers protocol. At 24 hpt medium was refreshed. At 48 hpt cells were infected with USUV at an MOI of 1 or transfected with pCAGGS-USUV-NS4A. At 24 h after the infection, supernatant, and cell lysates for protein and RNA analysis were collected. Cycloheximide (CHX) treatment was started at 24 hpt.

### Cycloheximide turnover assay

293T cells (in 12-wells plates) were transfected with pCAGGS-USUV-NS4A or with pCAGGS-USUV-NS4A and p62-FLAG using PEI 25KT. At 24 hpt the cells were treated with 30 µg/mL CHX. The cells were harvested at the indicated timepoints and subjected to western blot analysis.

### RNA isolation and RT-qPCR

After removal of the supernatant, cells were lysed in Tripure reagent (Roche). RNA was extracted from the lysates using 5PRIME Phase Lock Gel tubes (Quantabio) during the phase separation with chloroform. Next, RNA and GlycoBlue Coprecipitant (Invitrogen) were precipitated from the aqueous phase using isopropanol. Viral RNA copy numbers were determined by RT-qPCR using the TaqMan Fast Virus 1-step master mix (Thermo Fisher Scientific). To determine absolute copy numbers of USUV RNA, a standard curve of 10-fold serial dilutions of in vitro-transcribed RNA containing the RT-qPCR target sequence was taken along. RT-qPCR was performed in a CFX384 Touch Real-Time PCR Detection System (BioRad) using the following program: 5 min at 50 °C and 20 s at 95 °C followed by 45 cycles of 5 s at 95 °C, and 30 s at 60 °C. USUV intracellular copies were normalized to PGK1 mRNA levels, which were quantified by the TaqMan gene expression Assay Vic-MGB (assay ID Hs00943178_g1, Thermo Fisher Scientific, #4448491). The primers used for quantification of virus RNA copies are listed in table S2.

For the detection of host cell responses RT-qPCR was performed using iQ SYBR green Supermix (Biorad). The RNA was first reverse-transcribed into cDNA using the RevertAid H Minus reverse transcriptase (Thermo Scientific) and random hexamers. The cDNA samples were mixed with the iQ SYBR green Supermix and primers, and then run in a CFX384 Touch real-time PCR detection system using the following program: 3 min at 95 °C and 30 s at 60 °C followed by 40 cycles of 10 s at 95 °C, 10s at 60 °C and 30 s at 72 °C. Gene expression was normalized to expression of RPL13a, and quantified by the standard curve method. The primers used to quantify host mRNAs are listed in table S3.

### Statistical analysis

The data were plotted and analyzed using GraphPad Prism (version 9). An unpaired two-tailed Student’s t-test was performed to check for statistical significance. A p-value of < 0.05 was considered statistically significant.

## Results

### Mapping the USUV NS4A interactome

To better understand the role of USUV NS4A during infection, we investigated USUV NS4A-protein interactions in 293T cells using a proximity-based biotinylation strategy coupled to mass spectrometry [32]. We fused a biotin ligase, TurboID, to either the N-terminus or C-terminus of NS4A (creating NS4A-TurboID and TurboID-NS4A respectively), and transfected these constructs separately into 293T cells. Using this technique, interaction partners or proteins in close proximity to NS4A will get biotinylated by TurboID, while distal proteins will not. We then precipitated all the biotinylated proteins using NeutrAvidin beads, and confirmed by silver staining that the pulldown was successful (Fig. S1A). We identified the biotinylated proteome using mass spectrometry (Fig. 1B). A total of 2263 proteins, with each at least 2 peptides identified by mass spectrometry, were detected. From this set 391 proteins were significantly more abundant in both the NS4A-TurboID and TurboID-NS4A transfected cells compared to the negative control (Fig. S1B). MAVS, a known interactor of USUV NS4A [12], was in this list of enriched proteins, validating our approach. We performed Gene Ontology enrichment analysis on the significantly enriched interaction partners of USUV NS4A using g: Profiler (Fig. S1C). Protein binding was the most significantly enriched molecular function of the identified interactors (Fig. 1C). Most of the interaction partners are (partially) localized in the cytoplasm (Fig. 1D), which is not surprising since NS4A is present in the membrane of the ER. The most strongly enriched terms for the biological processes were intracellular transport and organelle organization (Fig. 1E). WNV NS4A is involved in the rearrangement of membranes [33], and based on the enrichment of proteins in organelle organization this might suggest USUV NS4A has a similar function. Interestingly, we found that autophagy, which for many orthoflaviviruses plays a key role during infection [18], was also one of the enriched biological processes. We found several autophagy-related proteins of which TMEM41B has been previously identified as a ZIKV NS4A interaction partner (Fig. 1F) [34]. Among the identified autophagy-related proteins, p62/SQSTM1 was the most abundant protein detected (Fig. 1F).

### USUV infection and NS4A overexpression induce autophagosomes

**Fig. 2:**
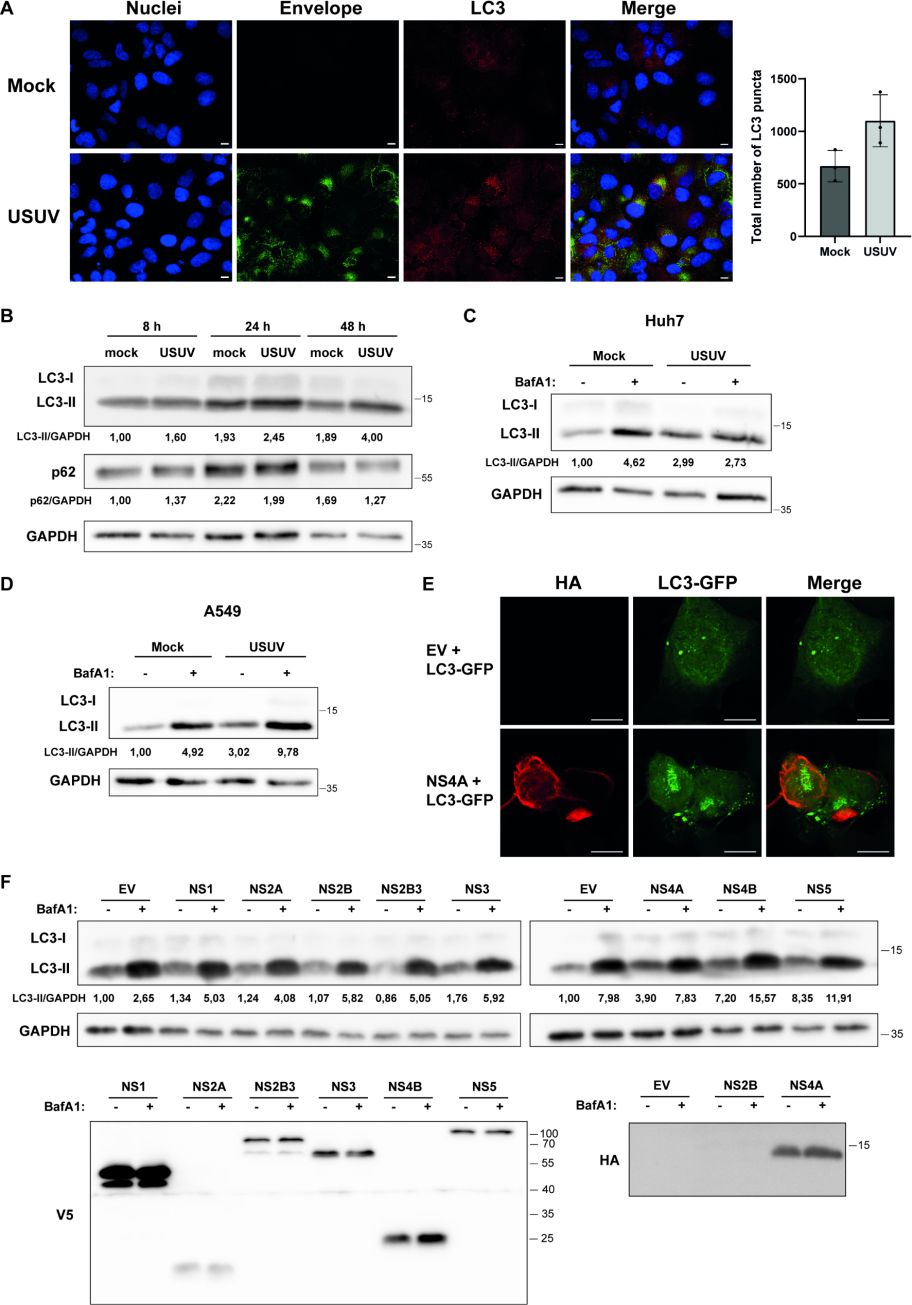
USUV infection and USUV NS4A overexpression induce the formation of autophagosomes. **A** Huh7 cells were infected with USUV (MOI = 1) and fixed at 24 hpi. Cells were stained for the orthoflavivirus envelope protein (green) and LC3 (red). Nuclei were visualized by Hoechst 33,258. Scale bars (white) in the bottom right corner represent 10 μm. Total number of LC3-puncta were determined in three independent images of the mock-infected and USUV-infected cells. Representative images are shown. **B** Huh7 cells were infected with USUV (MOI = 1) and protein lysates were collected at the indicated times. LC3, p62, and GAPDH levels were then analyzed by western blot. Numbers between the panels represent the band intensity ratio of LC3-II or p62 to GAPDH and are normalized to mock-infected cells at 8 hpi. **C-D** Huh7 cells (**C**) or A549 cells (**D**) were infected with USUV (MOI = 1) for 48 h. 6 h before harvest the cells were treated with 100 nM bafilomycin A1 (BafA1) or left untreated. Samples were immunoblotted for LC3 and GAPDH. Numbers between the panels represent the band intensity ratio of LC3-II to GAPDH and are normalized to the untreated mock-infected cells. **E** H1299 cells were co-transfected with LC3-GFP (green) and the plasmid encoding HA-tagged USUV NS4A or the empty vector control (EV). Cells were fixed at 24 hpt and stained for the HA tag (red). Scale bars (white) in the bottom right corner represent 10 μm. **F** 293T cells were transfected with different USUV protein expression plasmids, and treated with DMSO or BafA1 (100 nM) for 6 h before harvesting. At 24 hpt lysates were collected and immunoblotted for LC3, V5 (NS1, NS2A, NS2B3, NS3, NS4B, and NS5), HA (NS2B, and NS4A), and GAPDH. Numbers between the panels represent the band intensity ratio of LC3-II to GAPDH and are normalized to the empty vector (EV) transfected cells

Based on the interactome data, we hypothesized NS4A might play a role in modulating the autophagy pathway. Therefore we first studied whether USUV infection can trigger the autophagic response. We infected Huh7 cells with USUV and checked for LC3 expression by immunofluorescence and western blot analysis (Fig. 2A-B). Upon autophagy activation LC3-I will be converted to LC3-II by PE conjugation and incorporated in the autophagosome membrane, which makes LC3-II a good marker for the detection of autophagosomes by western blot [35]. The incorporation of LC3 into the autophagosome membrane can also be detected as puncta in immunofluorescence. USUV infection of Huh7 cells resulted in an increased number of LC3-puncta in the cells (Fig. 2A), indicating that USUV can induce autophagosomes. USUV infection of Huh7 cells also resulted in increased LC3-II levels at 48 h post infection (Fig. 2B), further supporting that autophagosomes are induced during USUV infection. Next, we checked whether the autophagic flux was increased after USUV infection, since increased LC3-II levels could be caused by either enhanced autophagic flux or by a block of lysosomal degradation. During activation of the full autophagy pathway p62 delivers substrates to the autophagosomes and as a consequence is degraded in the autophagosomes, thereby serving as a marker for autophagic flux. We only observed a minor decrease in p62 levels after USUV infection (Fig. 2B), which might indicate that autophagic flux is not enhanced during USUV infection. Autophagic flux can also be assessed by studying LC3-II levels after adding a late-stage autophagy inhibitor such as bafilomycin A1. When we treated mock-infected Huh7 cells with bafilomycin A1 there is a clear increase in LC3-II levels. However, when we infected the Huh7 cells with USUV and then treated with bafilomycin A1, we did not see an additional increase in LC3-II levels (Fig. 2C), which suggests that USUV blocks lysosomal degradation. Surprisingly though, when we performed the same experiment in A549 cells we did observe a clear increase in LC3-II levels in infected cells treated with bafilomycin A1 (Fig. 2D). This would indicate that the autophagic flux is enhanced by USUV in A549 cells. Overall, these results showed that USUV modulates the autophagy pathway, but depending on the cell-type different parts of the pathway might be affected. Next, we wanted to establish whether individual expression of USUV NS4A and other USUV nonstructural proteins (outside the context of infection) can induce autophagy. We transfected H1299 cells with the USUV nonstructural proteins and checked for LC3 puncta in the cell by immunofluorescence (Fig. 2E, and Fig. S2). Cells transfected with USUV NS4A showed a clear punctuated pattern of LC3 (Fig. 2E), while NS1 and NS4B transfected cells also were positive for LC3 puncta (Fig. S2). We then transfected 293T cells with the USUV nonstructural proteins and treated the cells with bafilomycin A1 to determine the autophagic flux after overexpression of individual viral proteins. We found that USUV NS4A, NS4B, and NS5 most strongly increased LC3-II levels (Fig. 2F). Together these results suggest that NS4A and NS4B expression alone are each sufficient to induce autophagosomes. Furthermore, treatment of transfected cells with BafA1 further increased LC3-II levels (Fig. 2F), which indicates that overexpression of these individual viral proteins lead to activation of the full autophagy flux. Overall, these results showed that NS4A can induce the autophagy pathway.

### The canonical autophagy pathway does not influence USUV replication

**Fig. 3:**
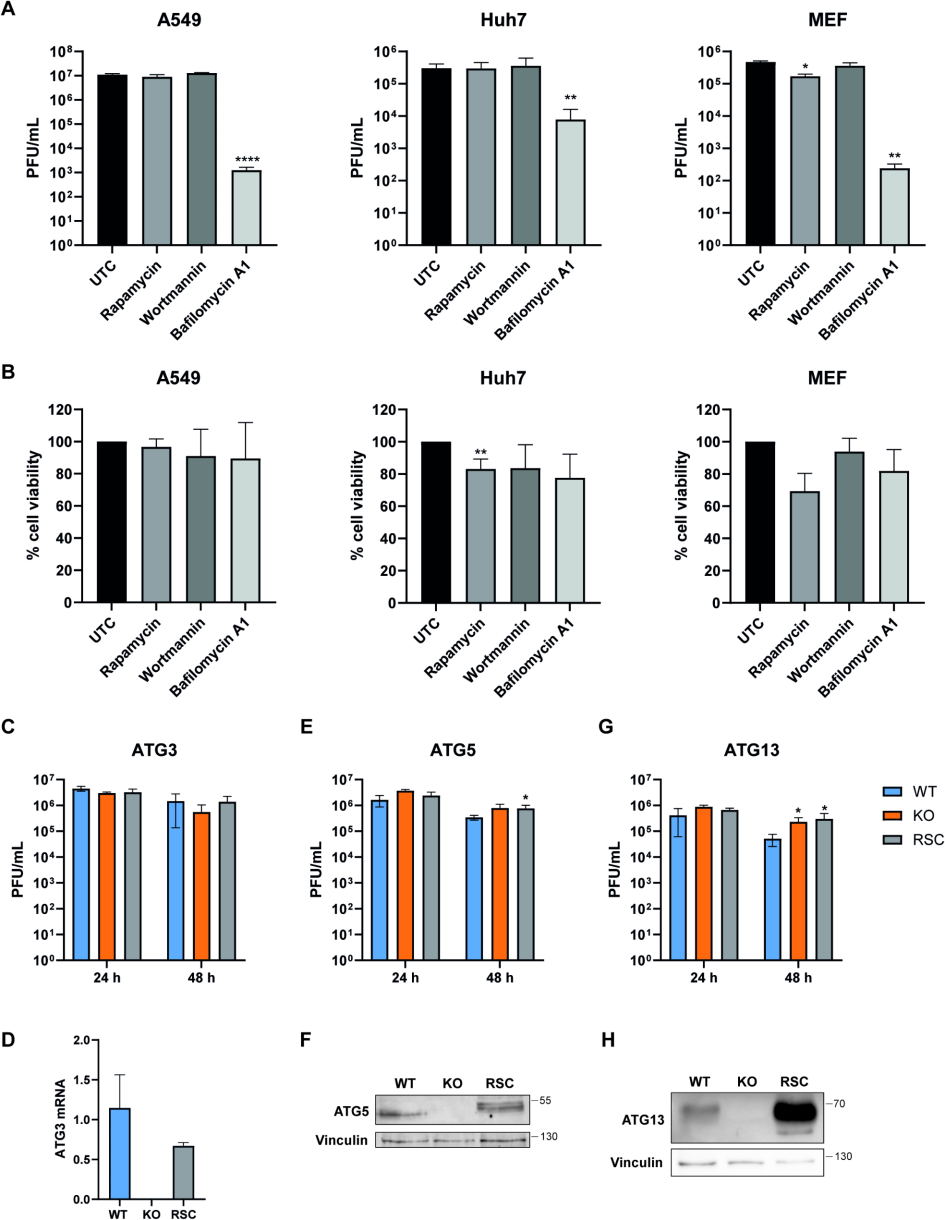
USUV replication is not influenced by the canonical autophagy pathway. **A-B** A549, Huh7, and MEF cells were infected with USUV (MOI = 1). After the 1-hour virus adsorption period, cells were treated with DMSO (negative control), rapamycin (1 μm), wortmannin (1 μm), or bafilomycin A1 (100nM). At 24 hpi, the supernatants were collected to determine virus titers by plaque assay (**A**) and the cell viability was measured by MTS (**B**). Means ± standard deviation of 2–3 independent experiments are shown and statistical significance relative to DMSO-treated cells is indicated (* *p* < 0.05, ** *p* < 0.01, **** *p* < 0.0001). **C-H** wildtype (WT) MEFs, MEFs with knockouts (KO) for the autophagy related genes ATG3 (**C-D**), ATG5 (**E-F**), and ATG13 (**G-H**), and the corresponding cells with reintroduced genes (RSC) were infected with USUV (MOI = 3). At 24 and 48 hpi, the supernatants were collected to determine virus titers by plaque assay. Means ± standard deviation of 2–4 independent experiments are shown and statistical significance relative to wildtype cells is indicated (* *p* < 0.05). **D**,** F**,** H** Knockout of the autophagy related genes and the reintroduction of the genes were validated by RT-qPCR (**D**) or western blot analysis (**F**,** H**)

Next we questioned whether the induction of the autophagy pathway might influence USUV replication either by restricting the virus or by facilitating virus replication. To this end, we analyzed the effect on USUV replication of several compounds commonly used to modulate the autophagy pathway. We induced the autophagy pathway in cells using rapamycin, while we inhibited the early steps of autophagy by using the PI3K inhibitor wortmannin or the late steps by using the lysosome inhibitor bafilomycin A1. We checked for the activity of the different compounds by assessing the formation of LC3 puncta in Huh7 cells (Fig. S3A). We then infected A549 cells, Huh7 cells and mouse embryonic fibroblasts (MEFs) with USUV, and treated them with rapamycin, wortmannin, or bafilomycin A1 for 24 h. We examined LC3-II levels after infection by western blot analysis, and assessed the effect of treatment on USUV replication. Surprisingly wortmannin treatment increased LC3-II levels in the mock-infected cell (Fig. S3B), suggesting that the treatment induced aspecific effects besides inhibiting the autophagy pathway. Neither rapamycin nor wortmannin affected USUV replication in the cell lines, except that rapamycin treatment of MEFs reduced USUV titers by 2,7-fold (Fig. 3A). However, rapamycin treatment of MEFs was toxic (cell viability < 80%), which might have caused this small reduction in USUV titers (Fig. 3B). Bafilomycin A1 resulted in almost 4-log reduction of the USUV titer in A549 cells, 3-log reduction in MEFs and a 39-fold reduction in the Huh7 cells (Fig. 3A). Since induction of autophagy by rapamycin and inhibition of the early steps of autophagy by wortmannin did not influence USUV replication, it is more likely that the decrease in USUV titers through bafilomycin A1 is unrelated to the autophagy pathway and is instead linked to other parts of the lysosomal degradation pathway. To further confirm whether autophagy influences USUV replication, we also infected cell lines containing knockouts of different autophagy related genes (ATG). We infected MEFs containing gene knockouts of ATG3, ATG5, or ATG13. ATG13 is part of the autophagy initiation complex, while ATG3 and ATG5 are both involved in the PE-conjugation of LC3 [18]. The ATG3 knockout resulted in a small and non-significant decrease in USUV replication at 48 h, which was rescued after reintroduction of the gene (Fig. 3C, D). The ATG5 knockout did not significantly change USUV replication at both time points (Fig. 3E, F). The ATG13 knockout resulted in a significant increase in USUV titers, however this could not be reversed by reintroducing the gene (Fig. 3G, H). Overall, these small and opposite effects of knockout of the ATG genes on USUV titers corroborate that autophagy in general does not influence USUV replication, but that potentially distinct parts of the autophagy pathway could play a role during USUV infection.

### P62/SQSTM1 interacts with NS4A and targets NS4A for degradation

**Fig. 4:**
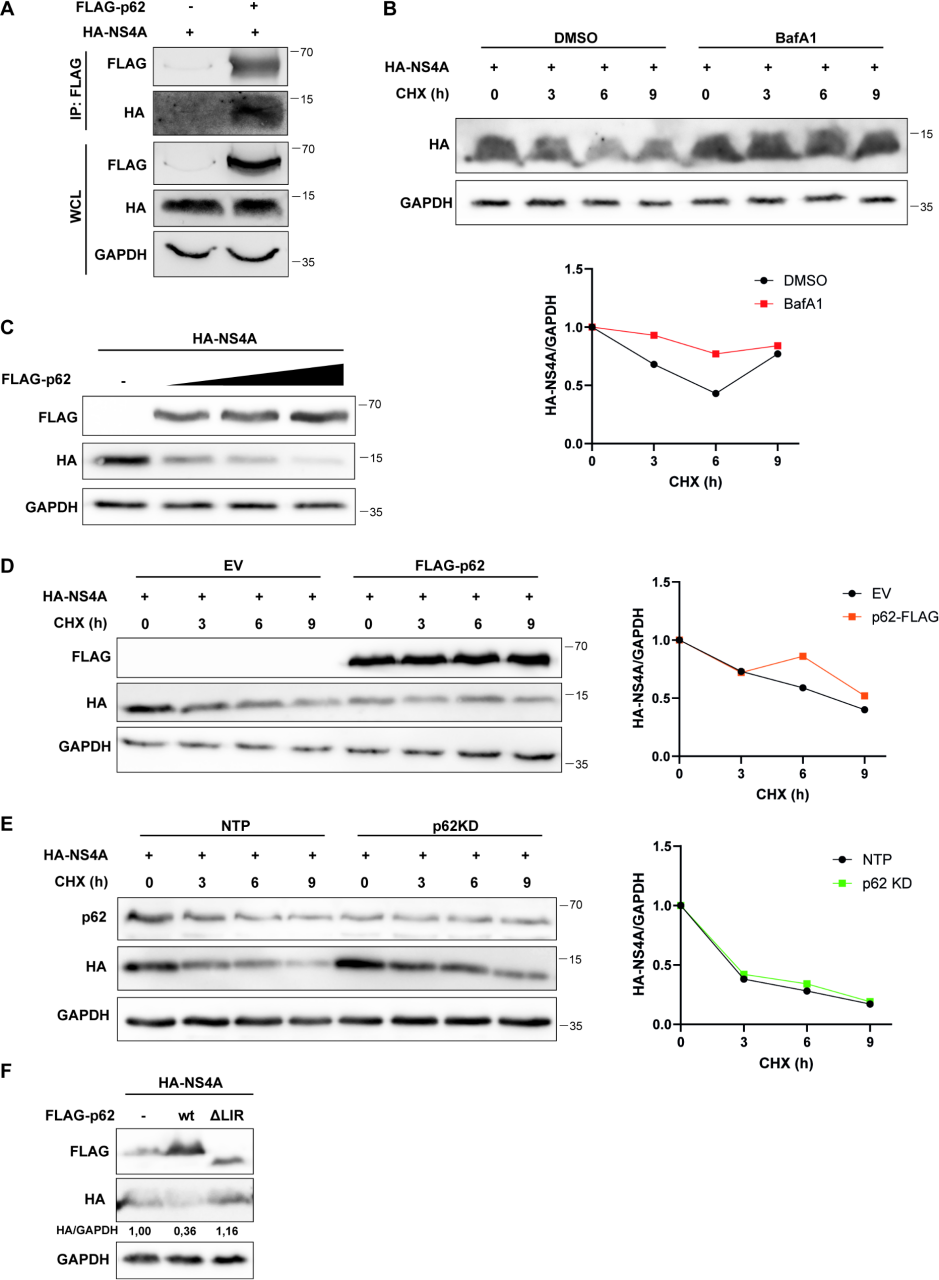
USUV NS4A is targeted by p62 for autophagic degradation. **A** 293T cells were co-transfected with expression plasmids for HA-tagged USUV NS4A and FLAG-tagged p62. At 24 hpt lysates were collected and immunoprecipitated with a FLAG antibody and the whole cell lysates and immunoprecipitates were analysed for the indicated proteins by western blot. **B** 293T cells were transfected with the USUV NS4A expression plasmid. 24 hpt cells were treated with 30 µg/mL cycloheximide (CHX) and with DMSO or 100nM bafilomycin A1 (BafA1). At the indicated times cells were harvested for immunoblotting with HA and GAPDH. Graph below the blots shows the band intensity ratio of HA to GAPDH and are normalized to the untreated cells (0 h). **C** 293T cells were transfected with the USUV NS4A expression plasmid and increasing amounts of the p62 plasmid (0.5 µg, 1 µg, and 1.5 µg). At 24 hpt cells were harvested and immunoblotted for HA, FLAG, and GAPDH. **D** 293T cells were co-transfected with the USUV NS4A and p62 expression plasmids. At 24 hpt cells were treated with 30 µg/mL CHX for the indicated times and harvested for immunoblotting with HA, FLAG, and GAPDH. Graph on the right shows the band intensity ratio of HA to GAPDH and are normalized to the untreated cells (0 h). **E** 293T cells were transfected with siRNA targeting p62 or the non-targeting pool (NTP). 48 h after the siRNA transfection, cells were transfected with the USUV NS4A expression plasmid. At 24 hpt cells were treated with 30 µg/mL CHX for the indicated times and harvested for immunoblotting with HA, p62, and GAPDH. Graph on the right shows the band intensity ratio of HA to GAPDH and are normalized to the untreated cells (0 h). **F** 293T cells were transfected with the USUV NS4A expression plasmid and plasmids expressing either the wildtype p62 (wt) or a mutant p62 lacking the LIR motif (ΔLIR). At 24 hpt cells were harvested and immunoblotted for HA, FLAG, and GAPDH. Numbers between the panels represent the band intensity ratio of HA to GAPDH and are normalized to the NS4A-transfected cells

We found p62, an autophagy-related cargo receptor, as one of the most abundant proteins in our mass spectrometry screen for interactors of USUV NS4A, and we therefore set out to better understand the role of p62 during USUV replication. We first performed a co-immunoprecipitation assay to confirm the interaction between p62 and USUV NS4A. NS4A coprecipitated with p62 after pull-down of p62 using a FLAG antibody, while no NS4A was detected when the empty vector was used (Fig. 4A). As mentioned p62 is a selective autophagy cargo receptor, which delivers proteins to the autophagosome [19]. Since NS4A and p62 interact, this might indicate that NS4A can be targeted for degradation by autophagy via p62. We first checked whether turnover of NS4A is influenced by autophagy. We transfected cells with USUV NS4A and then treated the cells with cycloheximide (CHX), an inhibitor of translation, at indicated times to study NS4A turnover. As expected USUV NS4A levels are decreasing over time after the CHX treatment (Fig. 4B), but if the cells are additionally treated with bafilomycin A1 this decline in NS4A levels is largely blocked. This shows that NS4A degradation is stimulated by autophagy. Next, we assessed whether this degradation of NS4A is mediated by p62. When an increasing amount of p62 was overexpressed in 293T cells together with NS4A, we observed a dose-dependent decrease in USUV NS4A levels (Fig. 4C), which suggests that p62 targets NS4A for degradation. To further assess this, we co-transfected p62 and NS4A in 293T cells and then treated the cells with CHX. We then observed lower levels of NS4A after CHX treatment when p62 was overexpressed, but the turnover time was not increased (Fig. 4D). When we performed a knockdown of p62 in the 293T cells using siRNA targeting p62, and then assessed NS4A levels, we observed higher levels of NS4A in the p62 knockdown cells (Fig. 4E). However, the rate of NS4A degradation was similar in the knockdown cells, suggesting that NS4A turnover is determined by other host factors as well. To determine whether the p62-mediated degradation of NS4A is via the autophagy pathway, we co-transfected 293T cells with NS4A and either wildtype p62 (wt) or a mutant p62 that lacks the LIR motif and therefore cannot bind to LC3 anymore (ΔLIR). We observed that p62-ΔLIR did not decrease NS4A levels in contrast to wt p62 (Fig. 4F), strongly suggesting that the decrease in NS4A levels after p62 overexpression depends on autophagy. Overall, these results showed that USUV NS4A can be targeted by p62 for degradation by the autophagy pathway.

### P62/SQSTM1 acts as an antiviral factor during USUV infection

**Fig. 5:**
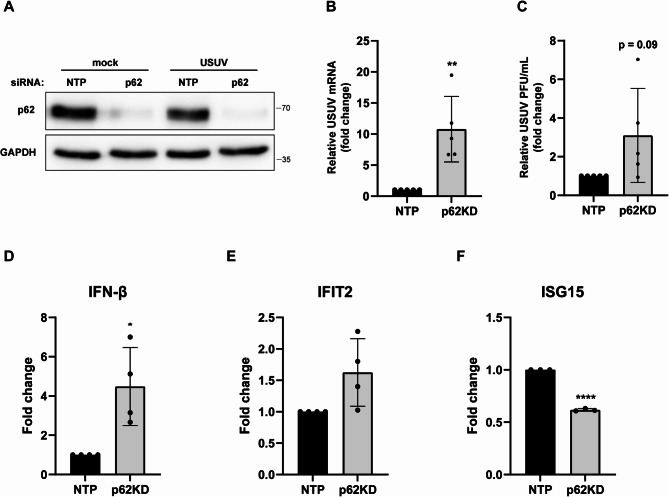
p62 has an antiviral effect during USUV replication and modulates the ISG response. A549 cells were transfected with siRNA targeting p62 or the non-targeting pool (NTP). At 48 hpt cells were infected with USUV (MOI = 1). At 24 hpi supernatants and lysates were collected. **A** Knockdown of p62 was confirmed by western blot analysis. **B-C** Viral mRNA copies (**B**) and infectious progeny (**C**) were determined by RT-qPCR and plaque assay, respectively. Means ± standard deviation of 5 independent experiments are shown and statistical significance relative to NTP-treated cells is indicated (** *p* < 0.01). **D-F** Total RNA was isolated from the lysates and IFN-β (**D**), IFIT2 (**E**), and ISG15 (**F**) mRNA levels were measured by RT-qPCR. Gene expression was normalized to RPL13a and the fold change versus NTP-infected cells calculated. Means ± standard deviation of 3–4 independent experiments are shown and statistical significance relative to NTP-treated cells is indicated (* *p* < 0.05, **** *p* < 0.0001)

Next, we examined the role of p62 during USUV replication. We transfected A549 cells with siRNA targeting p62 and then infected the cells with USUV. We confirmed successful knockdown of p62 by western blot analysis (Fig. 5A). The knockdown of p62 resulted in a significant increase of USUV intracellular RNA levels compared to the cells transfected with control siRNA (non-targeting pool; NTP) (Fig. 5B). A similar trend was observed when measuring the infectious titer, however the effect on infectious progeny varied between experiments (Fig. 5C). Taken together, this suggests that p62 acts as an antiviral factor during USUV replication, which is consistent with its role in the degradation of NS4A.

Besides the direct effect of p62 on viral proteins, it has also been shown that p62 can influence virus replication through modulation of the IFN response. p62 has been shown to increase the IFN response in context of several different virus infections [36, 37]. We observed higher levels of IFN-β after the knockdown of p62, indicating that p62 does not upregulate IFN production during USUV replication (Fig. 5D). However, expression of the IFN-stimulated gene (ISG) IFIT2 was only slightly increased (less than 2-fold upregulation), while ISG15 levels were even downregulated during infection after p62-knockdown (Fig. 5E-F). This might indicate that p62 can influence the ISG response during USUV infection.

## Discussion

Orthoflaviviruses depend on the host cell machinery for their replication, and at the same time have to interfere with the antiviral host defense systems to enable successful replication in the cell. This means that a complex network of interactions between viral and host proteins exists, which can also play an important role in viral pathogenesis [38]. USUV is an emerging orthoflavivirus, of which the interaction with the host cell machinery has not been characterized well, and the influence of host processes on the replication of USUV remains to be uncovered. We previously studied the interaction of USUV with the host IFN response, showing that USUV NS4A interacts with MAVS to inhibit the IFN response [12]. To further expand our knowledge on the functions of USUV NS4A, we mapped its interaction partners in the host cell. In this study, we identified 391 potential interaction partners of NS4A using proximity labeling coupled to mass spectrometry. This approach allowed us to identify both strongly and transiently interacting proteins of NS4A. Gene ontology enrichment analysis of the interaction partners revealed that USUV NS4A might interact with the cellular autophagic response.

The role of autophagy during orthoflavivirus replication has been extensively studied and earlier research showed that autophagy can act proviral, antiviral, or has no effect on replication, depending on the virus, virus strain, or cell-type used [18]. In case of DENV, many studies demonstrated that this virus can induce the autophagic response and use the autophagy pathway in diverse ways to promote its replication [18, 39]. The impact of autophagy on the replication of other orthoflaviviruses such as WNV is more complicated though, one study found that the induction of autophagy during WNV infection was dependent on the strain used. Moreover, pharmacological modulation of the autophagy pathway impacted WNV replication different depending on the WNV strain [40, 41]. Another study showed that WNV infection induced the autophagy pathway, but WNV replication was not influenced by autophagy activators and inhibitors [42], similar to our observations during USUV infection. Overall, these studies illustrate that the effect of the autophagy pathway on orthoflavivirus replication is not conserved, and it is therefore important to better characterize the interplay between USUV and autophagy. Blazquez et al. previously showed that USUV replication can induce an autophagic response in Vero cells [43], similar to the results we here found in A549 and Huh7 cells. However, when they treated cells with rapamycin or the autophagy inhibitor 3-methyladenine (3-MA) these researchers observed increased and decreased USUV titers, respectively, leading to the conclusion that the autophagy pathway promotes USUV replication. In contrast, we did not find any significant effect on USUV replication when treating the cells with autophagy modulators such as rapamycin. One difference between our studies was the USUV strain used. This might indicate that, similar to WNV, the interaction with the autophagy pathway is different depending on the USUV strain. We provided further evidence in this study that USUV does not rely on the autophagy machinery for its replication by infecting cell lines with knockouts of different autophagy related genes. Only small differences in USUV replication were observed after knockout of several ATG proteins. In addition, the titers slightly decreased in the ATG3 knockout cells, while the opposite was observed in the ATG5 and ATG13 knockout cells. Our results would therefore suggest that the canonical autophagy pathway as a whole does not influence USUV replication, but that distinct parts of the pathway may be involved in promoting or restricting USUV replication.

The most abundant hit among the autophagy related proteins from our screen was the selective autophagy receptor p62. P62 binds ubiquitinated proteins and delivers them to the autophagosome for degradation [44]. P62 has been shown to restrict the replication of different viruses including DENV and ZIKV [45–48]. The most common mechanism of action described for p62 has been the binding of viral proteins by p62 and the subsequent delivery of these viral proteins to the autophagosome for degradation. The interaction between USUV NS4A and p62 therefore made NS4A a likely target for degradation mediated by p62. Indeed we observed that NS4A degradation was stimulated by autophagy and p62 overexpression. Moreover, we observed increased USUV RNA levels and infectious progeny after knockdown of p62 demonstrating that p62 also acts to restrict USUV replication. It appears obvious that the antiviral effect of p62 is due to its role in promoting the degradation of NS4A. However, our results were obtained by expressing individual proteins and we were not able to confirm the degradation of NS4A during USUV infection due to the lack of a functional USUV NS4A antibody. Therefore, in the context of an USUV infection, it remains to be elucidated whether the targeting of NS4A and/or other mechanisms of action are involved in in restriction of USUV replication by p62.

Besides its role in selective autophagy, p62 also functions in many other signaling pathways. One well-characterized function of p62 is the induction of the NF-κB-mediated inflammatory response [49, 50]. In addition, a few reports indicate that p62 might also be involved in the positive regulation of the interferon response [36, 37]. Regulation of the innate immune response by p62 could therefore also contribute to its antiviral effect. We observed increased expression of IFN-β mRNA after infection of cells with a p62 knockdown, which could potentially be explained by the increase in viral RNA levels after knockdown leading to increased recognition by innate immune receptors. However, this would suggest p62 is not needed during USUV infection for an adequate IFN response. On the other hand, ISG15 mRNA levels were downregulated in the p62 knockdown cells after USUV infection, suggesting that some ISGs might be affected by p62 signaling. This could indicate that the antiviral functions of p62 are potentially due to a combination of effects, or may not be dependent on the autophagy pathway at all.

## Conclusions

In conclusion, this study showed that USUV infection and the overexpression of USUV NS4A can induce autophagy, although the canonical autophagy pathway as a whole does not appear to influence USUV replication. Furthermore, USUV NS4A was targeted for degradation through p62-mediated selective autophagy, uncovering a potential new mechanism of the host antiviral defense against USUV. In accordance, p62 knockdown enhanced USUV replication, potentially by acting through multiple pathways, revealing an antiviral function of p62 during USUV infection.

## Electronic supplementary material

Below is the link to the electronic supplementary material.

Supplementary Material 1

Supplementary Material 2

Supplementary Material 3

Supplementary Material 4

## Data Availability

The mass spectrometry proteomics data have been deposited to the ProteomeXchange Consortium via the PRIDE [51] partner repository with the dataset identifier PXD057492.

## Abbreviations

3-MA: 3-methyladenine
ATG: Autophagy-related protein/gene
BafA1: Bafilomycin A1
CHX: Cycloheximide
DENV: Dengue virus
ER: Endoplasmic reticulum
IFN: Interferon
ISG: Interferon-stimulated genes
LC3: Microtubule-associated protein 1 light chain 3
MEF: Mouse embryonic fibroblast
MOI: Multiplicity of infection
NS: Nonstructural
PE: Phosphatidylethanolamine
p62/SQSTM1: Sequestosome 1
Rapa: Rapamycin
USUV: Usutu virus
WNV: West Nile virus
ZIKV: Zika virus
