# Supplementary material for: Usutu virus NS4A induces autophagy and is targeted by the selective autophagy receptor p62/SQSTM1 for degradation
Source: Virol J. 2025 Apr 17;22:103. doi: 10.1186/s12985-025-02719-5 (PMC12004613; doi:10.1186/s12985-025-02719-5)
Supplement: Supplementary file 1 — Supplementary Material 1 [file 12985_2025_2719_MOESM1_ESM.docx]

# **Supporting information**

**Table S1** Primers for plasmid construction

| **Primer name** | **Primer sequence** **(5’-3’)** |
| --- | --- |
| TurboID-NS4A-F | TAAGCAGAATTCTGCATCAGCTGTGGGATTCCTTG |
| TurboID-NS4A-R | TGCTTACCCGGGTTATCGTTGCTTCTCAGGTTCAG |
| NS4A-TurboID-F | TAAGCACTCGAGATGTCAGCTGTGGGATTCCTTG |
| NS4A-TurboID-R | TGCTTAGAATTCGTCGTTGCTTCTCAGGTTCAG |
| P62-F | GTGGTGGAATTCTATGGCGTC |
| P62-R | TGCTTACTCGAGTTACTTGTCATCGTCGTCCTTGTAGTCTCGTCCTCGCAACGGCGGGGGATGCTTT |
| P62-ΔLIR-F1 | ATCCACTAGTCCAGTGTGGTG |
| P62-ΔLIR-R1 | CACTTCTTTTGACCCCTCGGACTCCAAGG |
| P62-ΔLIR-F2 | GTCCGAGGGGTCAAAAGAAGTGGACCCGTC |
| P62-ΔLIR-R2 | GTTTAAACGGGCCCTCTAGA |

**Table S2** RT-qPCR primers and probes for viral genomes

| **Primer name** | **Sequence** **(5’-3’)** |
| --- | --- |
| USUV-F | TCAGAAAAGACGTGCCAGAG |
| USUV-R | AAAGTCCTTCCGTCCTTCATG |
| USUV-Probe | CCTGAAAGTGGTTTGAGCAGAAAGGC |

**Table S3** RT-qPCR primers for cellular genes

| **Target** | **Forward primer** **(5’-3’)** | **Reverse primer (5’-3’)** |
| --- | --- | --- |
| hIFN-β | TGCTCCAGAACATCTTTG | GATGGTTTATCTGATGATAGAC |
| hIFIT2 | GGACCAAAGTCTAAATAGGG | GGCACTTGAATTCACATTG |
| hISG15 | TCCTGGTGAGGAATAACAAGGG | GTCAGCCAGAACAGGTCGTC |
| hRPL13a | AAGGTGGTGGTCGTACGCTGTG | CGGGAAGGGTTGGTGTTCATCC |
| mATG3 | TAAGGCTGACGCTGGAGGTGAA | GTGCTCAACTGTTAAAGGCTGCC |
| mRPL13a | AGGGGCAGGTTCTGGTATTG | TGTTGATGCCTTCACAGCGT |
