# Supplementary figures and images for: Usutu virus NS4A induces autophagy and is targeted by the selective autophagy receptor p62/SQSTM1 for degradation
Source: Virol J. 2025 Apr 17;22:103. doi: 10.1186/s12985-025-02719-5 (PMC12004613; doi:10.1186/s12985-025-02719-5)

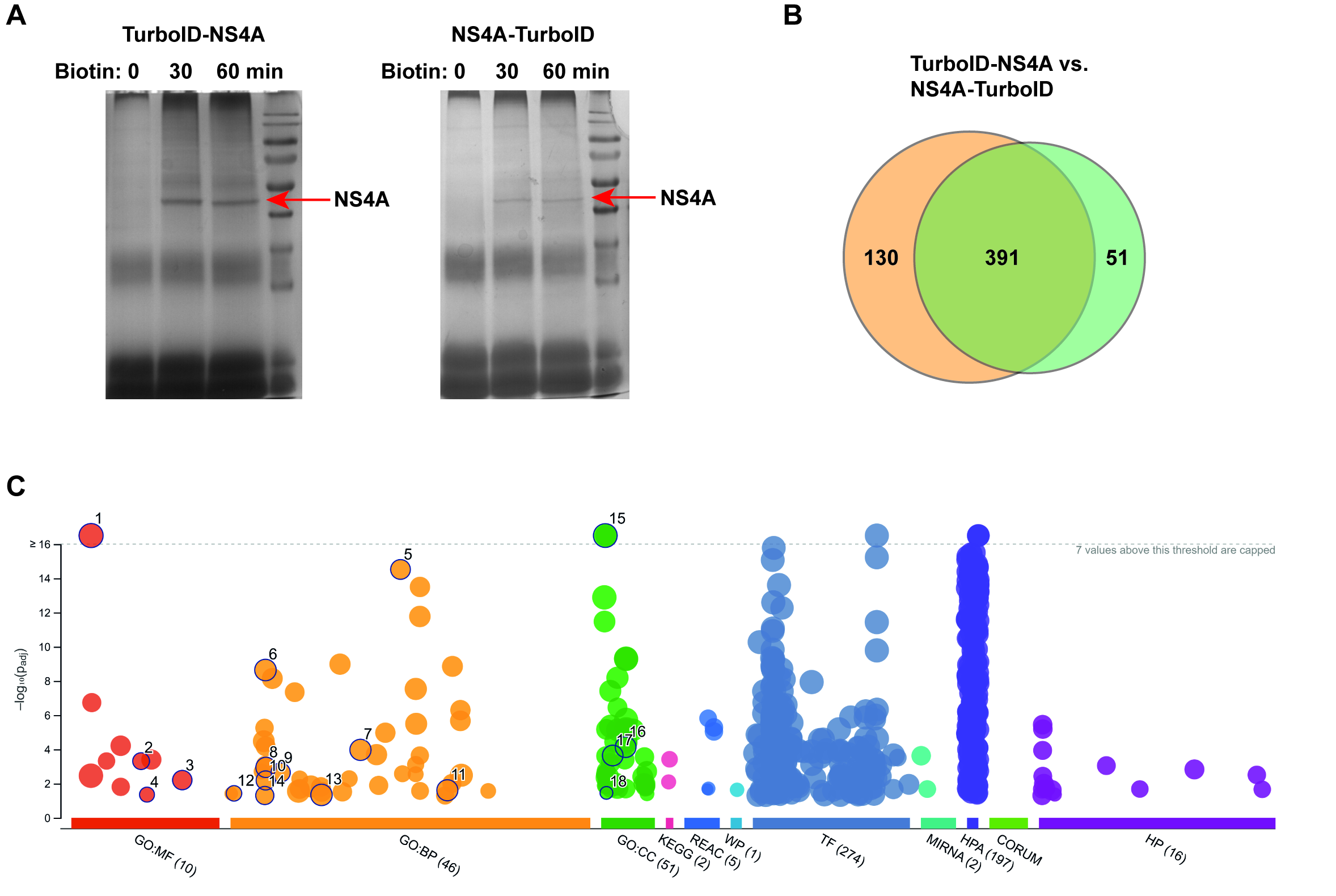

Supplement: Supplementary file 2 — Supplementary Material 2 [file 12985_2025_2719_MOESM2_ESM.tif]

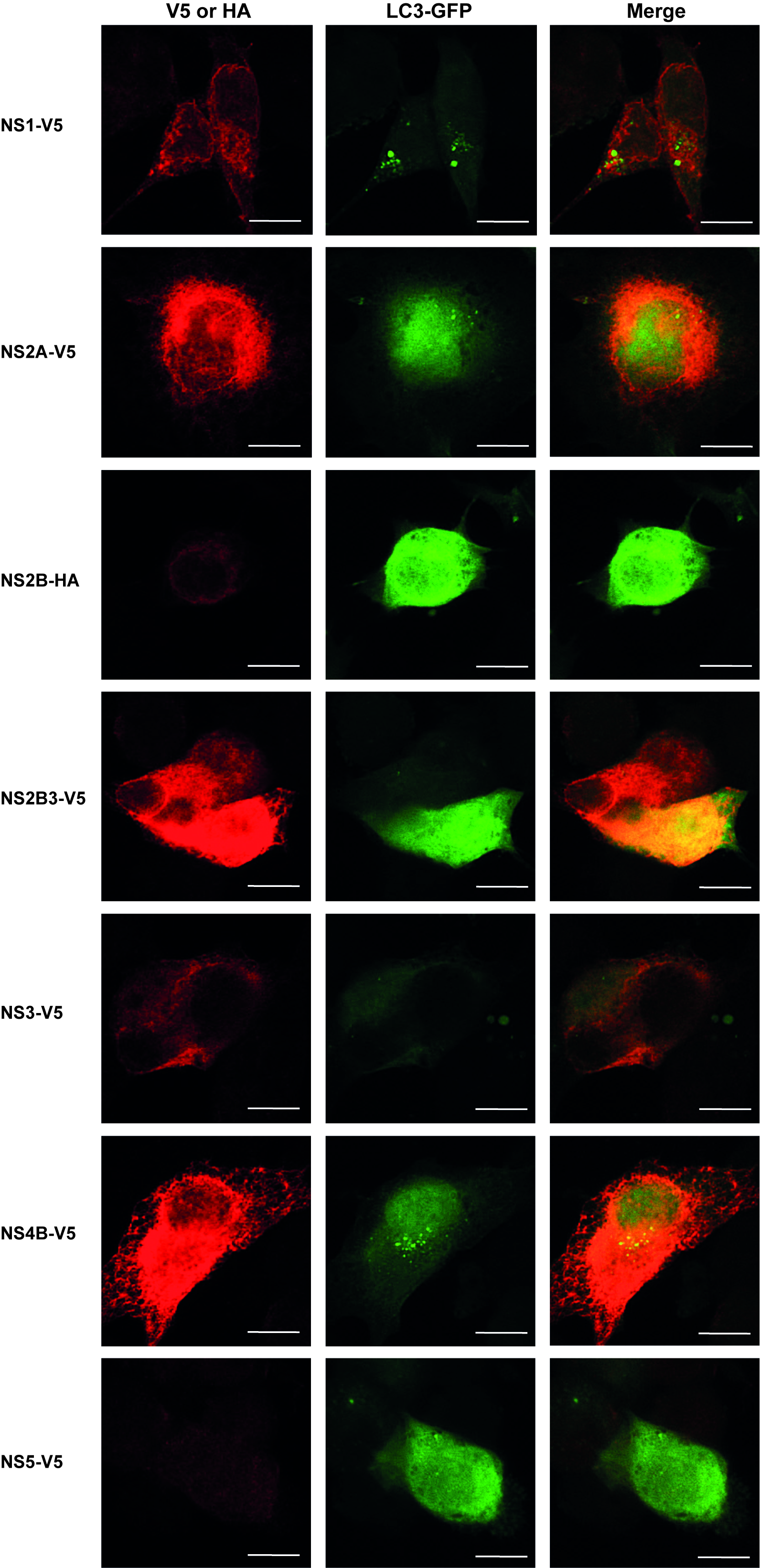

Supplement: Supplementary file 3 — Supplementary Material 3 [file 12985_2025_2719_MOESM3_ESM.tif]

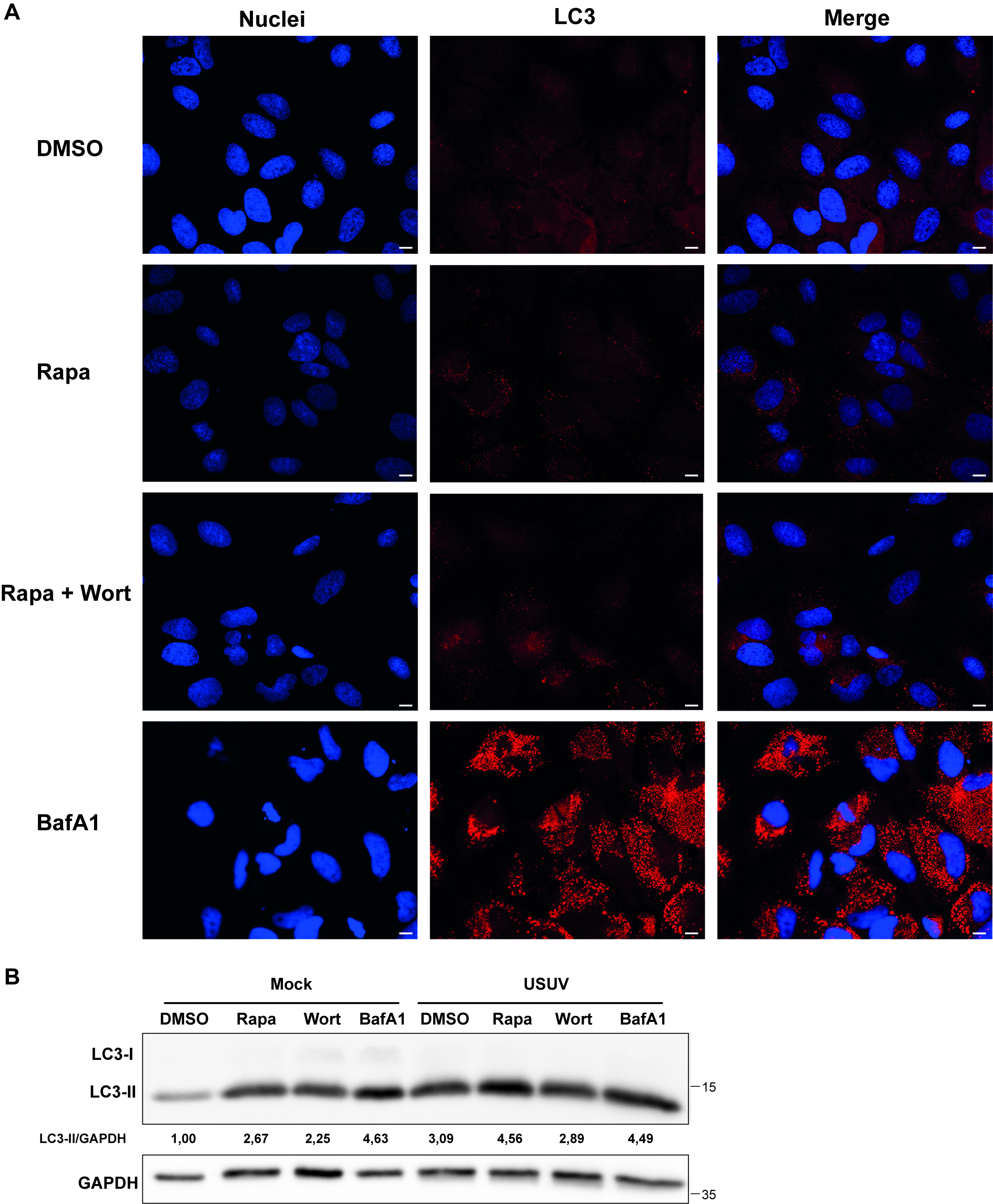

Supplement: Supplementary file 4 — Supplementary Material 4 [file 12985_2025_2719_MOESM4_ESM.tif]
